# Reliability of OMERACT Scoring System in Ultra-High Frequency Ultrasonography of Minor Salivary Glands: Inter-Rater Agreement Study

**DOI:** 10.3390/jimaging8040111

**Published:** 2022-04-15

**Authors:** Rossana Izzetti, Giovanni Fulvio, Marco Nisi, Stefano Gennai, Filippo Graziani

**Affiliations:** 1 Department of Surgical, Medical and Molecular Pathology and Critical Care Medicine, University of Pisa, 56126 Pisa, Italy; marco.nisi@unipi.it (M.N.); stefano.gennai@med.unipi.it (S.G.); filippo.graziani@unipi.it (F.G.); 2 Unit of Dentistry and Oral Surgery, University Hospital of Pisa, 56126 Pisa, Italy; 3 Department of Clinical and Experimental Medicine, University of Pisa, 56126 Pisa, Italy; giovanni.fulvio92@gmail.com; 4 Unit of Rheumatology, University Hospital of Pisa, 56126 Pisa, Italy

**Keywords:** salivary glands, diagnostic imaging, Sjögren’s syndrome diagnostic imaging, ultrasonography/ methods, ultra-high frequency ultrasound, reproducibility of results

## Abstract

Minor salivary gland ultra-high frequency ultrasonography (UHFUS) has recently been introduced for the evaluation of patients with suspected primary Sjögren’s Syndrome (pSS). At present, ultrasonographic assessment of major salivary glands is performed using the Outcome Measures in Rheumatology (OMERACT) scoring system. Previous reports have explored the possibility of applying the OMERACT scoring system to minor salivary glands UHFUS, with promising results. The aim of this study was to test the inter-reader concordance in the assignment of the OMERACT score to minor salivary gland UHFUS. The study was conducted on 170 minor salivary glands UHFUS scans of patients with suspected pSS. Three independent readers performed UHFUS image evaluation. Intraclass correlation coefficient (ICC) was employed to assess inter-reader reliability. Bland and Altman analysis was employed to test the agreement with a gold standard examiner. ICC values > 0.9 were found for scores 0 and 1, while score 2 and score 3 presented ICCs of 0.873 and 0.785, respectively. The measurements performed by the three examiners were in agreement with the gold standard examiner. According to these results, UHFUS interpretation showed good inter-observer reliability, suggesting that OMERACT score can be effectively used for the evaluation of glandular alterations, even for minor salivary glands.

## 1. Introduction

Primary Sjögren’s Syndrome (pSS) is a systemic autoimmune disease characterized by the progressive impairment of exocrine salivary and lachrymal glands. Glandular dysfunction results from the development of chronic lymphocytic infiltration in the glandular parenchyma [1]. The diagnostic work-up of pSS is often complex, as clinical presentation is extremely variable. Serologic, histopathologic, and functional tests are employed to detect signs of disease [2].

The 2016 American College of Rheumatology/European League Against Rheumatism (ACR/EULAR) classification criteria are reliably employed to diagnose the presence of pSS in patients with subjective ocular and/or oral dryness and/or a positive item on the EULAR Disease Activity Index for pSS (ESSDAI) [3,4]. Five items with different weight are identified and amount to diagnosis confirmation when an overall score ≥ 4 is obtained. Focal lymphocytic sialadenitis (focus score ≥ 1) and positive anti-SSA(Ro) serology contribute with three points each to the final score, while the other items (ocular staining score ≥ 5 (or van Bijsterveld score ≥ 4) on at least one eye; Schirmer test ≤ 5 mm/ 5 min on at least one eye; and unstimulated salivary flow rate ≤ 0.1 mL/min) are assigned a weight of 1 each [3]. 

It has been hypothesized that major salivary gland ultrasonography (SGUS) can improve the performance of the ACR/EULAR classification criteria [5]. The work by Jousse-Joulin et al. corroborated the hypothesis that abnormalities on SGUS can be associated with pSS diagnosis [6]. Additional evidence supports the improvement in terms of sensitivity, while not losing the specificity of the ACR/EULAR criteria by adding SGUS [7]. Salivary gland echostructure is evaluated with the Outcome Measures in Rheumatology (OMERACT) scoring system, which recognizes four degrees (score 0–3) of progressively severe glandular alteration [8,9].

Ultrasonography is increasingly employed in the investigation of various conditions of the oral cavity, due to the unique characteristics that make this diagnostic imaging modality a valid alternative to ionizing radiations [10]. Recently, a novel ultrasonographic technique, ultra-high frequency ultrasonography (UHFUS) has been introduced as a potential adjunctive tool in pSS diagnostic work-up, for the evaluation of labial minor salivary glands [11,12,13,14]. In our previous work, the OMERACT scoring system was applied to the evaluation of minor salivary glands. Preliminary assessment on the feasibility of UHFUS in patients with suspected pSS highlighted that intraoral minor salivary gland UHFUS showed a good correlation with histopathology, and can improve bioptic sampling [12,13]. 

The aim of the present study was to evaluate the intra- and inter-reader reliability of minor salivary glands UHFUS, as evaluated with the OMERACT scoring system.

## 2. Materials and Methods

### 2.1. Study Design and Registration

The study involved the evaluation of minor salivary glands UHFUS scans belonging to patients with suspected pSS and who completed the diagnostic work-up. Cases with incomplete data or who did not obtain a final diagnosis were excluded. All patients whose cases were utilized signed an informed consent form. The study protocol was approved by the Institutional Review Board of the University Hospital of Pisa (CEAVNO, protocol number 14540) and was conducted in accordance with the principles outlined in the Declaration of Helsinki. The manuscript was prepared according to the EQUATOR research reporting guidelines [15].

### 2.2. UHFUS Scoring System of Minor Salivary Glands

**Figure 1:**
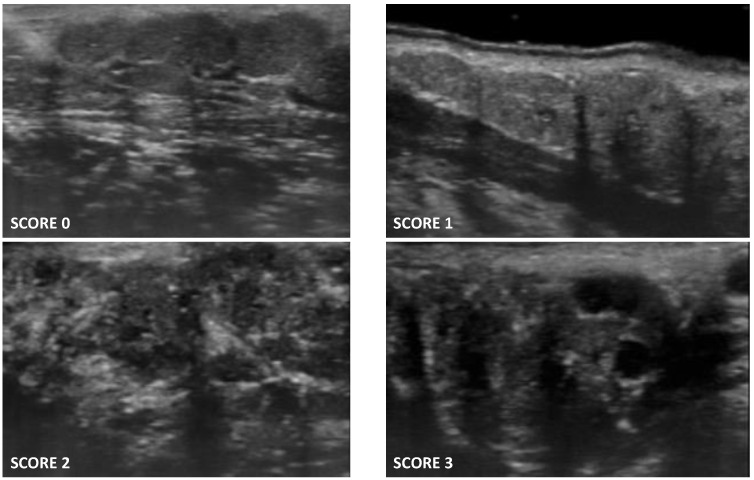
UHFUS scoring system of minor salivary glands.

The scoring system employed for the evaluation of minor salivary glands UHFUS was described in detail in the original report [12]. Briefly, minor salivary gland echostructure was assessed using the OMERACT scoring system, which is normally employed for major salivary gland evaluation. In particular, four degrees of glandular alteration were detected (Figure 1):

- Score 0: normal glandular parenchyma in the absence of alterations;
- Score 1: presence of fine echogenicity in the absence of clear alterations, or slight, diffuse glandular hypoechogenicity, mild glandular alteration;
- Score 2: presence of focal hypoechoic areas, but partial conservation of normal glandular parenchyma, moderate glandular alteration;
- Score 3: diffuse presence of hypoechoic areas in the absence of normal glandular parenchyma, or the presence of glandular fibrosis, severe glandular alteration.

### 2.3. UHFUS Scans Acquisition Protocol

A single examiner performed all UHFUS scans under standard conditions using a standardized acquisition protocol, previously described in the original report [12]. Minor salivary gland UHFUS examination was performed with a 70-MHz probe (UHF 70, Vevo MD equipment, FUJIFILM VisualSonics Inc., Toronto, Canada) with the following technical features: nominal frequency 52 MHz, bandwidth 29–71 MHz, axial resolution 30 μm, lateral resolution 65 μm, maximum depth 10.0 mm, maximum image width 9.7 mm, maximum image depth 10.0 mm, and focal depth 5 mm. The probe was covered with a disposable sterile cover for the management of cross-infection. Sterile ultrasound gel was applied to facilitate ultrasound beam transmission. Lower lip mucosa was entirely scanned, and axial and longitudinal B-mode acquisitions were obtained. All the scans were conducted under a standardized pre-set. The scans were saved and exported in DICOM format and were processed with Horos software (Version 3.3.6, https://horosproject.org (accessed on 15 February 2022)).

### 2.4. Evaluation of Inter-Rater Reliability of UHFUS Scoring System of Minor Salivary Glands

Three examiners participated in this reliability study. Prior to the study beginning, the UHFUS scoring system of minor salivary glands described in the original report [12] was explained to the examiners. Clarifications on the tasks or the classification system were provided to the examiners on request.

The examiners then received B-mode videos for each study case of suspected pSS, with the dataset including 170 progressively numbered cases. B-mode videos were prepared by an examiner not taking part in the reliability assessment. All videos maintained the original standardized 1:1 ratio and the original image quality. The brightness, contrast, and color of the images were not adjusted. The examiners were blinded to the study cases and were not provided with any additional information, in order to further reduce the risk of bias. The highest score detected on the exam was assigned to each case, as described in previous reports on OMERACT score assignment to ultrasound images of major salivary glands [16]. A gold standard examiner, who contributed to the original study on UHFUS scoring system of minor salivary glands, rated all the included cases. Comparison with a gold standard examiner was set as the benchmark to test the reliability of the classification.

### 2.5. Statistical Analysis

Intraclass correlation coefficient (ICC) was employed to assess the inter-reader reliability. The Bland and Altman test was used to verify the possible agreement between with the gold standard examiner [17]. All statistical analyses were performed with SPSS software (Statistical Package for Social Sciences, version 26.0-SPSS, Chicago, IL, USA). *p*-value was set as *p* < 0.05.

## 3. Results

### 3.1. Sample Characteristics

One hundred and seventy minor salivary gland UHFUS scans were analyzed in total. The study sample was composed of 159 females (93.5%; mean age 53.2, SD 12.6) and 11 males (6.5%; mean age 57.6, SD 11.9). A diagnosis of pSS was confirmed in 88 cases, while the remaining 82 patients were diagnosed with sicca syndrome. No differences in terms of age and gender distribution were found between pSS and sicca patients.

### 3.2. Image Analysis

Higher ICC values were encountered for scores 0 and 1 (0.938 and 0.953, respectively). In Score 2, 0.873 ICC was found. Score 3 presented the lowest ICC, with a value of 0.785 (Table 1).

### 3.3. Analysis of Agreement with the Gold Standard Examiner 

**Figure 2:**
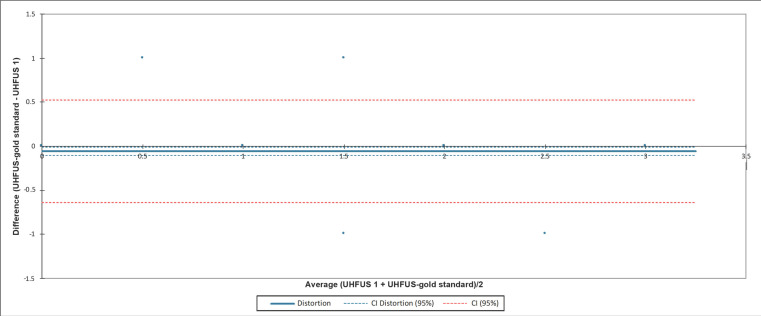
Bland-Altman test evaluating the agreement between the first reader (UHFUS 1) and the gold standard examiner (UHFUS-gold standard). The 95% confidence interval (CI) of the distortion was (−0.101; 0.013).

**Figure 3:**
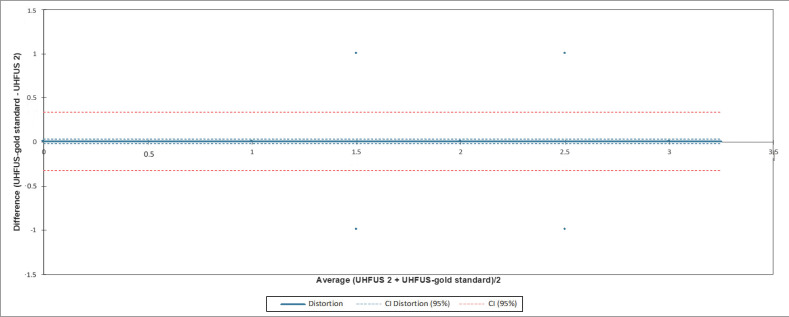
Bland-Altman test evaluating the agreement between the second reader (UHFUS 2) and gold standard examiner (UHFUS-gold standard). The 95% confidence interval (CI) of the distortion was (−0.019; 0.031).

**Figure 4:**
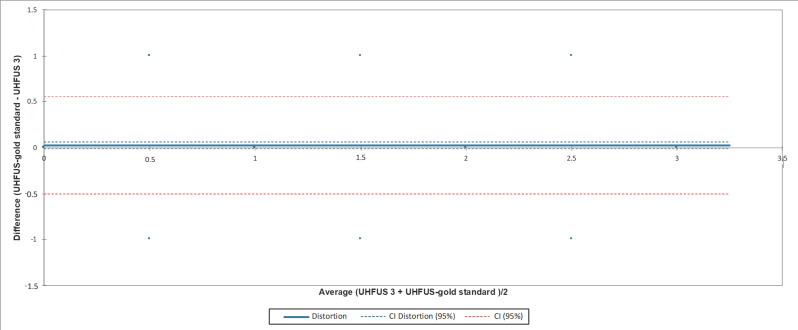
Bland-Altman test evaluating the agreement between the third reader (UHFUS 3) and gold standard examiner (UHFUS-gold standard). The 95% confidence interval (CI) of the distortion was (−0.012; 0.069).

The confidence interval (CI) of the distortion included zero when comparing the measurements of the three examiners with the gold standard examiner. In Figure 2, Figure 3 and Figure 4, the agreement between readers compared to the gold standard is reported. 

## 4. Discussion

The application of the OMERACT scoring system to UHFUS of minor salivary glands showed good inter-reader reliability. Interestingly, a higher concordance was detected for scores 0 and 1; thus, suggesting that the OMERACT scoring system can discriminate between normal glands and incipient glandular alteration. The lowest concordance was detected for score 3, presumably related to the tendency to underestimate glandular alteration.

The role of ultrasonography in the diagnostic work-up of pSS has been repeatedly discussed in the literature. However, the lack of consensus on the evaluation of salivary gland elementary lesions and, subsequently, on the scoring system have hindered the inclusion of SGUS in pSS classification criteria [18]. 

The pathologic changes occurring in the course of pSS, which eventually result in glandular damage, can be detected with SGUS as hypo/anechoic areas and hyperechoic bands [19,20,21]. In 1992, De Vita and coll. [19] proposed an ultrasonographic score assigning points to the different degrees of glandular inhomogeneity. Hocevar et al. [20] devised a scoring system evaluating five ultrasonographic parameters in total, namely echogenicity, inhomogeneity, number of hypoechogenic areas, hyperechoic reflections, and clearness of the borders of the salivary gland. The reliability of this system was tested in the study by Delli et al. [21], where discrepancies between observers were found when assessing the severity of ultrasound findings. The authors eventually concluded that the ultrasonographic follow-up of pSS progression and/or the evaluation of treatment efficacy should be performed by the same sonographer at every time point [21]. The systematic review by Jousse-Joulin et al. [22] recognized that despite the heterogeneity of SGUS scoring systems reported in the literature, SGUS presented a higher sensitivity and specificity in pSS diagnosis compared with other imaging techniques.

The issue of the reliability of ultrasonographic scores has been discussed in previous literature with regards to SGUS [6,23]. Specifically, two main limitations were recognized: (i) the testing of the reliability without a comprehensive scoring, and (ii) the moderate and fair inter-reader reliability in the evaluation of the homogeneity of the parotid and submandibular glands, respectively [6]. Nevertheless, it should be highlighted that adding SGUS to the ACR/EULAR criteria can increase the classification criteria sensitivity by 5–10% [6]. In order to overcome the limitations of the previous evaluation, the OMERACT SGUS subtask force implemented the definitions of normal US appearance and abnormalities seen in patients with suspected or confirmed pSS [23]. Importantly, the OMERACT scoring system for major salivary glands showed good inter- and intra-reader reliability of greyscale lesions and anechoic/hypoechoic foci using a semi-quantitative evaluation [23]. These results were further confirmed in the work by Zabotti et al. [24], suggesting that even less-expert sonographers could reliably evaluate major salivary glands on ultrasonography if adequately instructed. 

The evidence on the reliability of the OMERACT scoring system in SGUS was the rationale behind the application of these classification criteria to UHFUS of minor salivary glands [12]. UHFUS is increasingly being applied in several medical fields, especially for the investigation of small anatomical structures, due to the high resolution provided [25,26]. Previous evidence from our group supports the identification of a high correspondence between UHFUS and the histology of minor salivary glands; thus, suggesting a potential role for the technique in ruling out the presence of pSS [12,13]. However, we deemed it necessary to assess the intra- and inter-reader reliability, to increase the generalizability of our previous results. Overall, a high concordance was found between readers for scores 0–2, while score 3 presented the lowest concordance rates. Importantly, the examiners showed ICC values > 0.9 in scores 0 and 1. The examiners were able to discriminate between the absence of disease and incipient glandular alteration, allowing speculating on the potential use of UHFUS in detecting early-stage pSS patients, when irreversible glandular alteration has not yet occurred. In early stages of disease, which include the preclinical stage and the asymptomatic pSS stage, the therapeutic interventions aim at preserving exocrine glandular function or inhibiting autoimmune attack, to delay pSS progression [27]. Therefore, the performance of an early diagnosis appears of utmost importance for disease control.

However, the present study has some limitations. First, the study was conducted on previously recorded scans and, due to technical issues, UHFUS performance is likely to introduce systematic and non-systematic errors in global assessment. Second, the OMERACT scoring system is usually employed for the evaluation of major salivary glands, and the highest score from the three compartments was chosen. Although in the previous literature the application of the most affected salivary gland criterion has been employed for score assignment, further assessment and adjustment to the classification may be advised. Nevertheless, if these results are confirmed, UHFUS has the potential to become a valuable tool for the screening of patients with suspected pSS.

## 5. Conclusions

The evaluation of minor salivary gland UHFUS with the OMERACT scoring system showed good inter-observer reliability. These results appear extremely promising and pave the way for further investigations of the use of UHFUS to improve the morphological assessment of minor salivary glands in pSS.

## Figures and Tables

**Table 1:** Concordance rates classified per score assignment.

OMERACT Scoring System	ICC	95% Cis (Lower, Upper Bound)
Score 0	0.938	(0.912, 0.964)
Score 1	0.953	(0.928, 0.979)
Score 2	0.873	(0.853, 0.902)
Score 3	0.785	(0.756, 0.847)

## Data Availability

Data is contained within the article.
